# Efficacy and safety of inotuzumab ozogamicin and its combination therapies in acute lymphoblastic leukemia: a systematic review and meta-analysis

**DOI:** 10.3389/fonc.2025.1613777

**Published:** 2025-11-04

**Authors:** Changtao Lao, Jiange Wen, Yuezhen Wen

**Affiliations:** ^1^ Department of Hematopathology, Peking University Shenzhen Hospital, Shenzhen, China; ^2^ Nursing Department, Shenshan Medical Center, Sun Yat-sen Memorial Hospital, Sun Yat-sen University, Shanwei, China

**Keywords:** inotuzumab ozogamicin, acute lymphoblastic leukemia, meta-analysis, systemic review, efficacy

## Abstract

**Background:**

Although treatment for acute lymphoblastic leukemia (ALL) has advanced considerably, adults with relapsed or refractory (R/R) disease continue to face a grave prognosis. Inotuzumab ozogamicin (InO), a CD22-directed antibody–drug conjugate, represents a promising development for B-cell ALL. This systematic review and meta-analysis aims to define the precise efficacy and safety profile of InO-based therapies—both monotherapy and combination regimens—in adults with newly diagnosed and R/R ALL.

**Methods:**

A systematic literature search was conducted in PubMed, Web of Science, Embase, the Cochrane Library, and clinical trial registries through 2 December 2024. The primary outcomes were overall response (OR), complete remission (CR), minimal residual disease (MRD) negativity, overall survival (OS), and the rate of stem cell transplantation (SCT). Secondary outcomes comprised adverse events (AEs) and relapse.

**Results:**

The meta-analysis included 1 randomized controlled trial (RCT), 14 single-arm studies, and 1 clinical trial, encompassing 1,068 patients. The pooled efficacy outcomes were as follows: OR rate:89.0% [95% confidence interval (CI): 85.8%–92.2%, 95% prediction interval (PI): 1.2%–99.9%; *I* ^2^ = 90.1%, *p*<0.001], CR rate: 70.5% (95% CI: 58.6%–82.5%, 95% PI: 3.3%–76.9%; *I* ^2^ = 94.0%, *p*<0.001), MRD− rate: 84.6% (95% CI: 79.5%–89.6%, 95% PI: 0.4%–99.8%; *I* ^2^ = 80.9%, *p*<0.001), 1-year OS rate: 61.7% (95% CI: 44.9%–78.5%; *I* ^2^ = 94.1%, *p*<0.001), 2-year OS rate: 51.4% (95% CI: 32.2%–70.7%; *I* ^2^ = 93.8%, *p*<0.001), 3-year OS rate: 46.9% (95% CI: 22.5%–71.4%, 95%; *I* ^2^ = 95.2%, *p*<0.001), 5-year OS rate: 44.9% (95% CI: 39.2%–50.5%, 95%; *I* ^2^ = 0.0%, *p* =0.482), SCT rate: 27.5% (95% CI: 16.6%–38.4%, 95% PI: 1.2%–79.4%; *I* ^2^ = 95.2%, *p*<0.001), relapse rate: 23.6% (95% CI: 16.6%–30.6%, 95% PI: 16.6%–99.6%; *I* ^2^ = 78.2%, *p*<0.001), and incidence of veno-occlusive disease (VOD): 6.2% (95% CI: 3.8%–8.6%, 95% PI: 6.6%–54.5%; *I* ^2^ = 68.0%, *p*<0.001).

**Conclusion:**

InO demonstrates significant efficacy and a manageable safety profile in adult patients with ALL, supporting its use as a viable therapeutic option. Further randomized studies are needed to validate these findings.

**Systematic review registration:**

https://www.crd.york.ac.uk/prospero/, identifier CRD42024619042.

## 1 Introduction

Acute lymphoblastic leukemia (ALL) is an aggressive hematologic malignancy that originates from the clonal expansion of immature B- or T-lymphoid precursors in the bone marrow (1). This malignant proliferation disrupts normal hematopoiesis, leading to cytopenias (anemia, neutropenia, and thrombocytopenia), recurrent infections, and hemorrhagic diathesis. Without timely therapeutic intervention, ALL often progresses rapidly to fatal complications. Significant progress has been made in the treatment of ALL, particularly in pediatric patients, who have achieved high cure rates and favorable long-term prognoses with contemporary treatment regimens. However, adult patients with relapsed or refractory (R/R) ALL continue to experience poor clinical outcomes, underscoring the need for novel therapeutic approaches (2). Although conventional chemotherapy remains the first-line treatment due to its well-established protocols and cost-effectiveness, it is limited by treatment-related toxicities and primary resistance (3). Hematopoietic stem cell transplantation (HSCT) remains the only potentially curative approach for patients with R/R ALL, but achieving complete remission (CR) and minimal residual disease (MRD)-negative status before transplantation is crucial to optimize transplant outcomes (4). To improve clinical outcomes and survival in patients with ALL, novel therapies such as immunotherapies (5, 6) and targeted therapies (7, 8) have emerged, offering superior efficacy and more manageable toxicity profiles than conventional chemotherapy. However, these therapies are often restricted to certain subtypes of ALL with specific molecular alterations (e.g., BCR-ABL1 and FLT3-ITD), limiting their applicability (9). In contrast, immunotherapies targeting universally expressed antigens, such as CD19 and CD22 (present in over 90% of B-cell ALL cases), provide a more broadly applicable treatment approach (10). Therefore, there is an urgent need to develop therapies that offer enhanced efficacy and tolerability for the broader population of patients with ALL.

Inotuzumab ozogamicin (InO) is a CD22-directed antibody–drug conjugate (ADC) composed of a humanized anti-CD22 monoclonal antibody linked to a cytotoxic calicheamicin derivative. Upon binding to CD22 on B-cell membranes, InO undergoes rapid internalization through receptor-mediated endocytosis. Inside the cell, the conjugate releases its cytotoxic payload, inducing DNA double-strand breaks and triggering apoptosis in malignant B cells (11). As a targeted immunotherapeutic agent, InO has become an important treatment option for R/R ALL, with its clinical application increasingly extending to combination regimens that include conventional chemotherapy or immune checkpoint inhibitors. Despite encouraging clinical results, the efficacy and safety profiles of InO-based combination therapies remain insufficiently characterized, as current evidence is fragmented and lacks comprehensive synthesis. Although prior studies have clarified the therapeutic outcomes of InO monotherapy (12), the synergistic potential and cumulative toxicities of its combination regimens require further rigorous evaluation. This systematic review and meta-analysis integrated data from adult patients with ALL across different disease statuses. Recognizing the biological and therapeutic distinctions between newly diagnosed and R/R ALL, direct pooling of these populations could introduce substantial clinical heterogeneity. Therefore, in addition to assessing the overall efficacy and safety of InO, subgroup analyses were conducted according to treatment strategy (monotherapy *vs*. combination therapy) and disease status (newly diagnosed *vs*. relapsed/refractory) to generate evidence applicable to specific clinical contexts. The findings of this study aim to inform clinical decision-making and identify priorities for future research.

## 2 Article type

This systematic review and meta-analysis was rigorously conducted following the Preferred Reporting Items for Systematic Reviews and Meta-Analyses (PRISMA) guidelines (13, 14) and adhered to the methodological standards prescribed in the Cochrane Handbook for Systematic Reviews of Interventions.

### 2.1 Search strategy

We performed a comprehensive search of the following databases: PubMed, Embase, Cochrane Library, Web of Science, and ClinicalTrials.gov. The search was conducted from the inception of each database to 2 December 2024. The key terms used in the search were “Acute Lymphoblastic Leukemia” AND “Inotuzumab Ozogamicin,” with the following PubMed-specific search string: (“acute lymphoblastic leukemia” OR “precursor cell lymphoblastic leukemia lymphoma”) AND (“Inotuzumab Ozogamicin” OR “CMC-544” OR “Besponsa”). No restrictions were applied regarding language, geographic region, ethnicity, or age. To ensure comprehensive coverage, we additionally hand-searched the reference lists of relevant review articles and primary studies. The complete search strategy is detailed in **Supplementary Material 1**.

### 2.2 Inclusion and exclusion criteria

Inclusion criteria.

(1) Randomized controlled trials (RCTs), single-arm, studies and conference abstracts; (2) studies involving patients ≥18 years old with confirmed ALL or its subtypes; (3) studies of InO either as monotherapy or in combination regimens; and (4) studies reporting at least one of the following efficacy or safety outcomes were included: overall response (OR), CR, MRD negativity (MRD−), overall survival (OS), stem cell transplantation (SCT), adverse events (AEs), and relapse.

Exclusion criteria.

(1) Studies reporting outcomes from mixed populations or disease cohorts; (2) studies primarily evaluating other drugs where only a subset of patients received InO; and (3) reviews, commentaries, case reports, studies with incomplete data, and cellular or animal studies.

### 2.3 Data extraction

Two investigators independently extracted data from the included studies. Any discrepancies between the reviewers were resolved through discussion with a third author. The following characteristics were systematically recorded for each included study: first author’s name, publication year, study design, sample size, treatment regimen, follow-up duration, disease status, patient age, and outcome measures. Efficacy outcomes including OR, CR, and MRD; survival outcome (OS); safety outcomes (AEs); transplantation outcomes (SCT); and relapse outcomes were documented using a predesigned data collection form (**Supplementary Material 6**).

### 2.4 Quality assessment

For RCTs, the risk of bias was assessed using the modified Jadad scale (15). This scale evaluates four domains: (1) random sequence generation (0–2 points), (2) allocation concealment (0–2 points), (3) blinding (0–2 points), and (4) withdrawals and dropouts (0–1 point). The total score ranges from 0 to 7, with studies scoring 1–3 classified as low quality and those scoring 4–7 classified as high quality. For single-arm studies, methodological quality was evaluated using the Methodological Index for Non-Randomized Studies (MINORS) (16). The criteria included the following: clearly stated study objectives, consecutive patient inclusion, prospective data collection, appropriate endpoints, unbiased endpoint assessment, adequate follow-up, dropout rate ≤5%, and *a priori* sample size calculation. Each of the eight items was scored from 0 to 2, yielding a total score ranging from 0 to 16. Studies were classified as low quality (score <9), moderate quality (score 9–12), or high quality (score >12).

### 2.5 Statistical analysis

All statistical analyses were performed using STATA version 18.0. A random-effects model was applied for all meta-analyses to account for anticipated clinical and methodological heterogeneity across studies. Sensitivity analyses were conducted to explore potential sources of heterogeneity and evaluate the robustness of pooled estimates. For outcomes demonstrating substantial heterogeneity (*I* ^2^ > 50%), the Freeman–Tukey double arcsine transformation was employed to stabilize variances. Subgroup analyses were performed according to treatment regimen and disease status to assess variations in the efficacy and safety of InO. Publication bias was evaluated using funnel plots and Egger’s regression test when 10 or more studies were available. When significant publication bias was detected (*p*<0.05), the trim-and-fill method was applied to adjust for its potential impact.

## 3 Results

### 3.1 Study selection and characteristics

**Figure 1:**
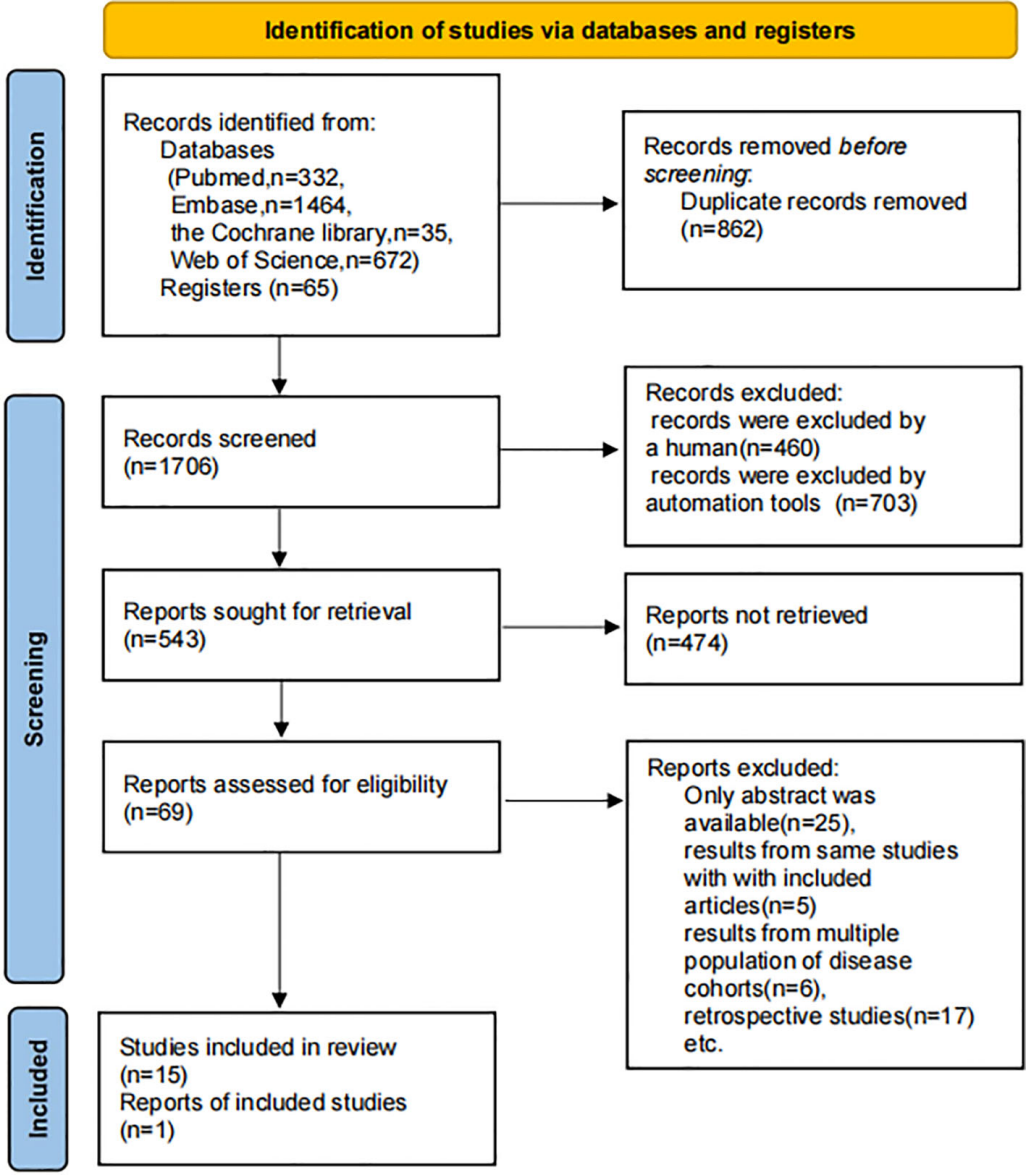
Flowchart of the selection process of studies.

A total of 2,503 references and 65 NCT-registered trials were initially identified. After removing duplicates and screening titles and abstracts, followed by full-text assessment, 16 studies were ultimately included in the analysis. These comprised 1 clinical trial (NCT03677596) (17), 1 RCT (18), and 14 single-arm studies (including 6 full-text publications and 8 conference abstracts) (19–32). The study selection process is illustrated in **Figure 1**. The included studies collectively enrolled 1,068 patients, with ages ranging from 18 to 87 years, including 492 newly diagnosed cases and 576 R/R cases. Treatment regimens included InO monotherapy (five studies), InO combined with chemotherapy (five studies), InO combined with blinatumomab (one study), and InO combined with chemotherapy, with or without blinatumomab (five studies). Detailed characteristics are provided in **Supplementary Material 6**.

### 3.2 Quality assessment

Among the included studies, one RCT (18) was classified as high quality based on a score of 4 points on the modified Jadad scale (**Table 1A**). The remaining 14 single-arm studies and conference abstracts (19–32) were evaluated using the MINORS index, yielding quality scores ranging from 12 to 15 (**Table 1B**). The results of the methodological quality assessment indicated that all included studies met the criteria set for this meta-analysis.

**Table 1:** Quality assessment of included studies.

A. Modified Jadad scale for included RCT studies.									
Study	Randomization		Concealment of allocation		Double blinding		Withdrawals and dropouts		Total
Kantarjian et al. (18)	2		0		1		1		4
B. MINORS index for included non-randomized studies.									
Study	I	II	III	IV	V	VI	VII	VIII	Total
De Angelo et al. (19)	2	2	2	2	0	2	2	2	14
Kopmar et al. (20)	2	2	2	2	0	2	2	2	14
Marconi et al. (25)	1	2	2	2	0	1	2	2	12
Chevallier et al. (21)	2	2	2	2	0	2	2	2	14
Nasr et al. (26)	1	2	2	2	0	2	2	2	13
Kantarjian et al. (22)	2	2	2	2	0	2	2	2	14
Jabbour et al. (23)	2	2	2	2	0	2	2	2	14
Wieduwilt et al. (27)	1	2	2	2	0	2	2	2	13
Stelljes et al. (24)	2	2	2	2	0	2	2	2	14
Advani et al. (28)	1	2	2	2	0	1	2	2	12
Jen et al. (29)	1	2	2	2	0	2	2	2	13
Nasnas et al. (30)	1	2	2	2	0	2	2	2	13
Rafei et al. (31)	1	2	2	2	0	2	2	2	13
Short et al. (32)	1	2	2	2	0	2	2	2	13

### 3.3 Quality assessment

#### 3.3.1 Overall response

**Figure 2:**
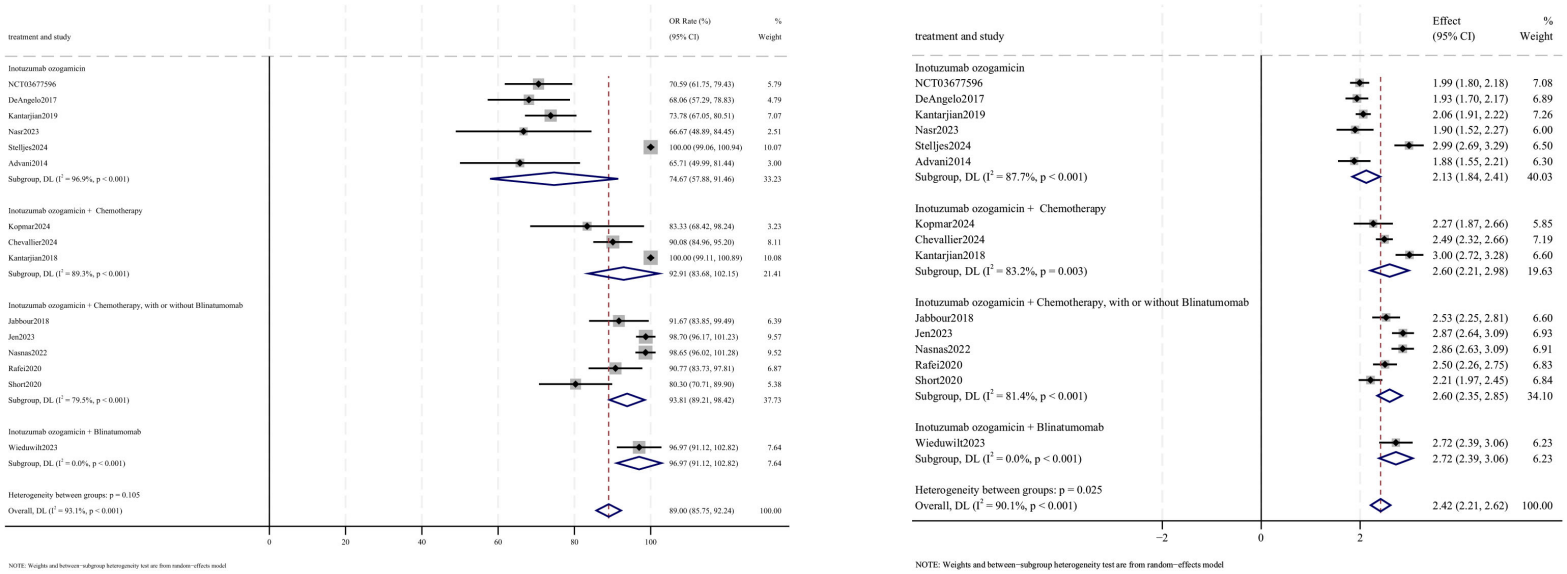
Forest map for subgroup analysis of treatment regimens in OR.

**Figure 3:**
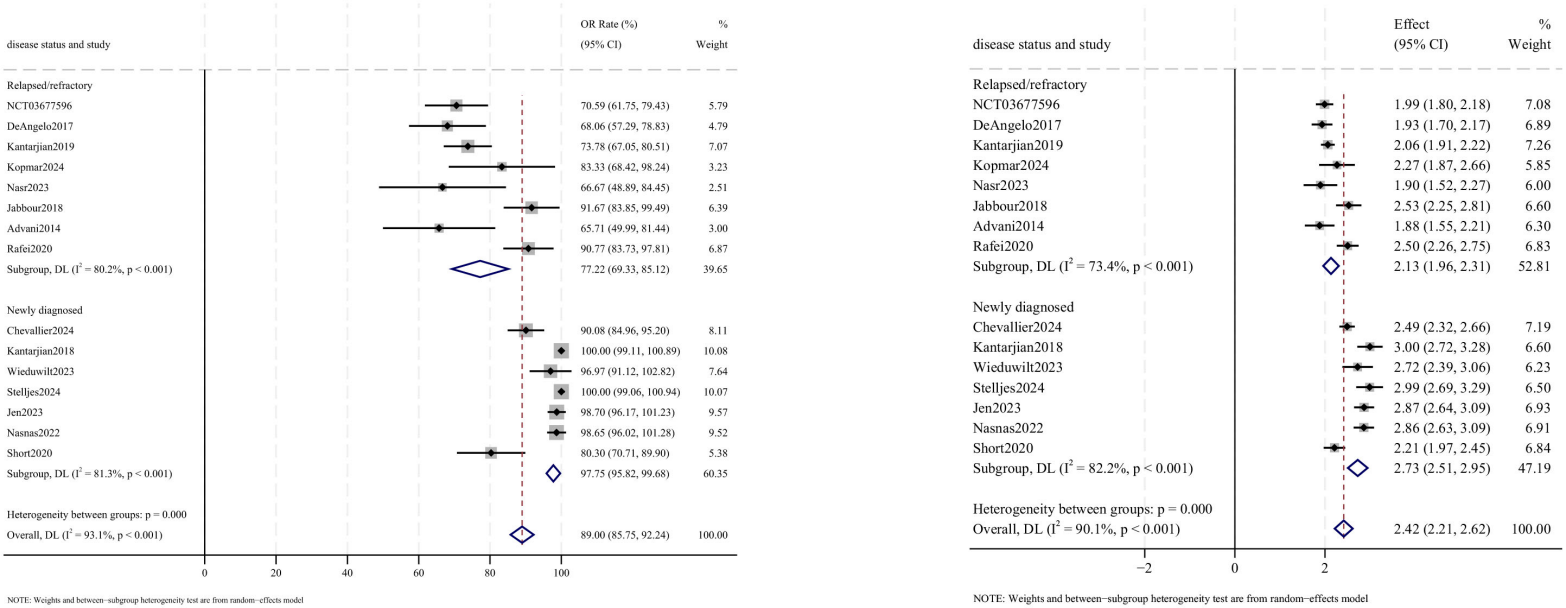
Forest map for subgroup analysis of disease status in OR.

OR outcomes were reported in 15 studies, with analysis using a random-effects model yielding a pooled OR rate of 89.0% [95% confidence interval (CI): 85.8%–92.2%, 95% prediction interval (PI): 1.2%–99.9%; *I* ^2^ = 90.1%, *p*<0.001] (**Figures 2**, **3**). Subgroup analyses revealed two findings: (1) InO combined with chemotherapy, with or without blinatumomab showed the highest OR rate at 93.8% (95% CI: 89.2%–98.4%; *I* ^2^ = 79.5%, *p*<0.001); (2) newly diagnosed patients with ALL demonstrated superior OR rates of 97.8% (95% CI: 95.8%–99.7%; *I* ^2^ = 81.3%, *p*<0.001). Sensitivity analysis employing the leave-one-out method confirmed that all effect sizes remained within the 95% CIs, indicating robust result stability (**Supplementary Material 2**). Both the funnel plot (**Supplementary Material 3**) and Egger’s linear regression test (*p* =0.452, **Supplementary Material 4**) showed no significant evidence of publication bias.

#### 3.3.2 Complete remission

**Figure 4:**
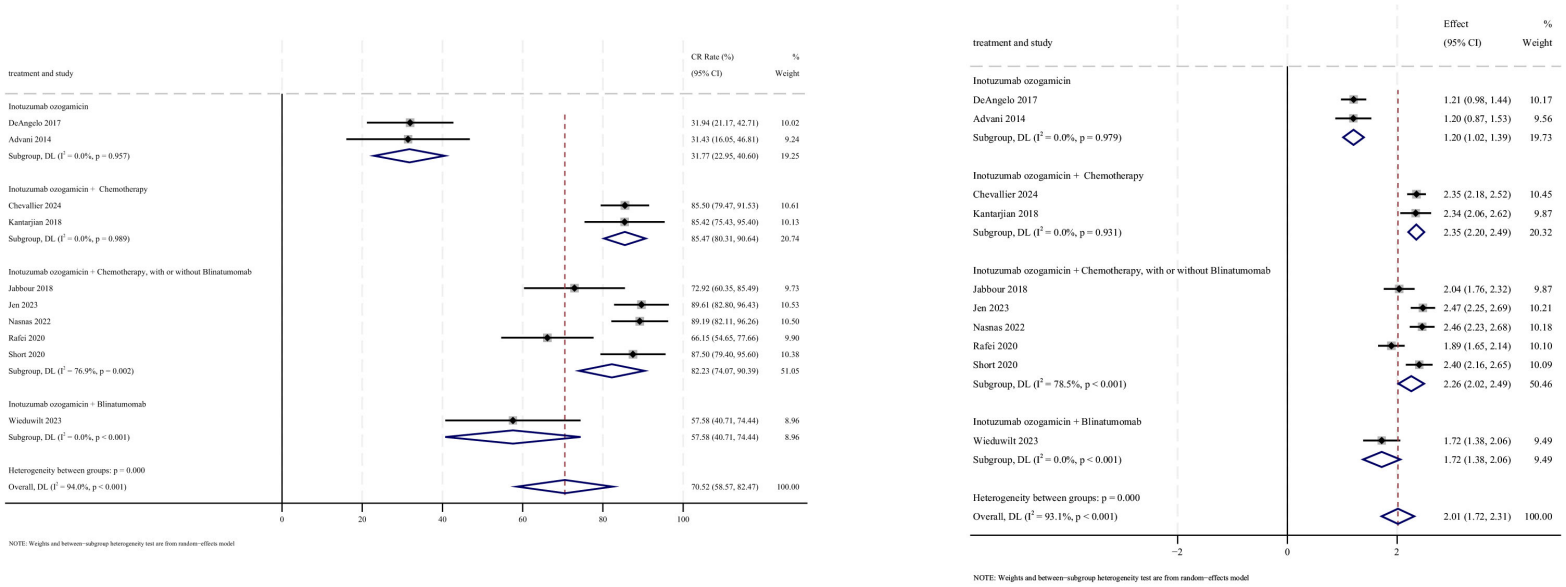
Forest map for subgroup analysis of treatment regimens in CR.

**Figure 5:**
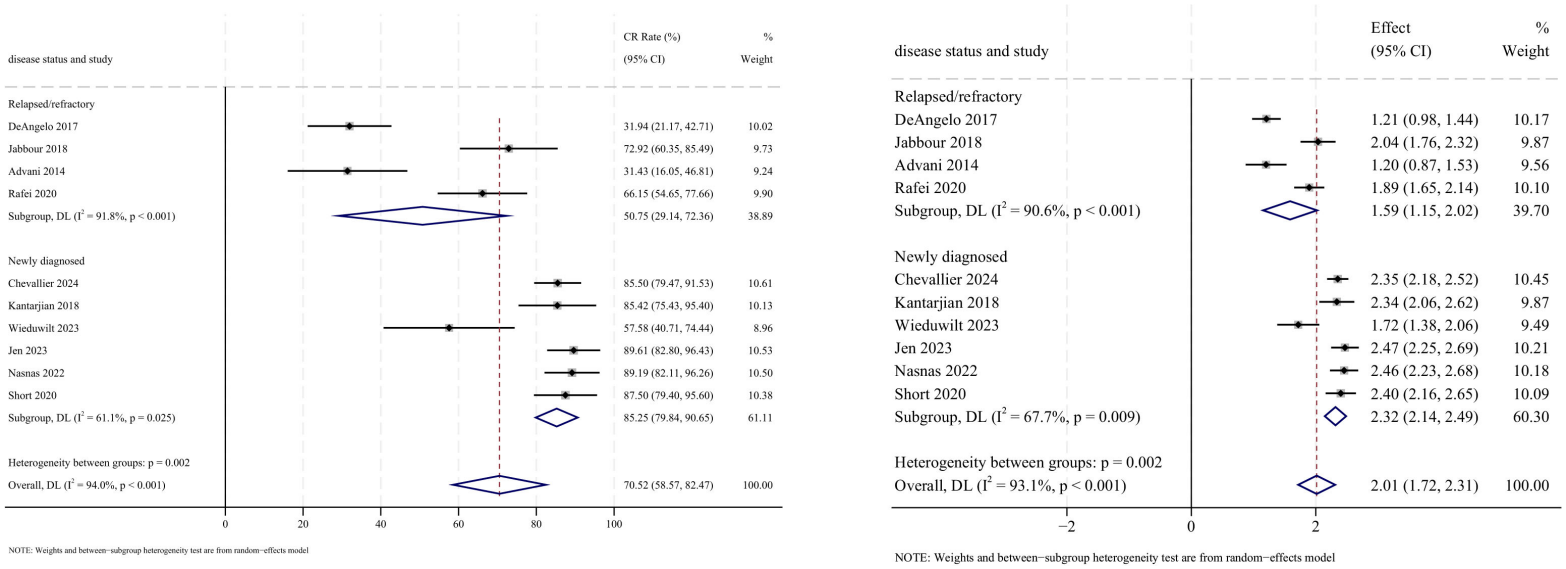
Forest map for subgroup analysis of disease status in CR.

CR outcomes were reported in 10 studies, with analysis using a random-effects model yielding a pooled CR rate of 70.5% (95% CI: 58.6%–82.5%, 95% PI: 3.3%–76.9%; *I* ^2^ = 94.0%, *p*<0.001). Subgroup analyses revealed two findings: (1) InO combination with chemotherapy regimen demonstrated the highest CR rate at 85.5% (95% CI: 80.3%–90.6%; *I* ^2^ = 0.0%, *p* =0.989) (**Figures 4**, **5**); (2) newly diagnosed patients with ALL showed superior CR rates of 85.3% (95% CI: 79.8%–90.7%; *I* ^2^ = 61.1%, *P*=0.025). Sensitivity analysis employing the leave-one-out method confirmed that all effect sizes remained within the 95% CIs, indicating robust stability of the findings (**Supplementary Material 2**). Both the funnel plot (**Supplementary Material 3**) and Egger’s linear regression test (*p* =0.191, **Supplementary Material 4**) showed no significant evidence of publication bias.

#### 3.3.3 Minimal residual disease-negative rate

**Figure 6:**
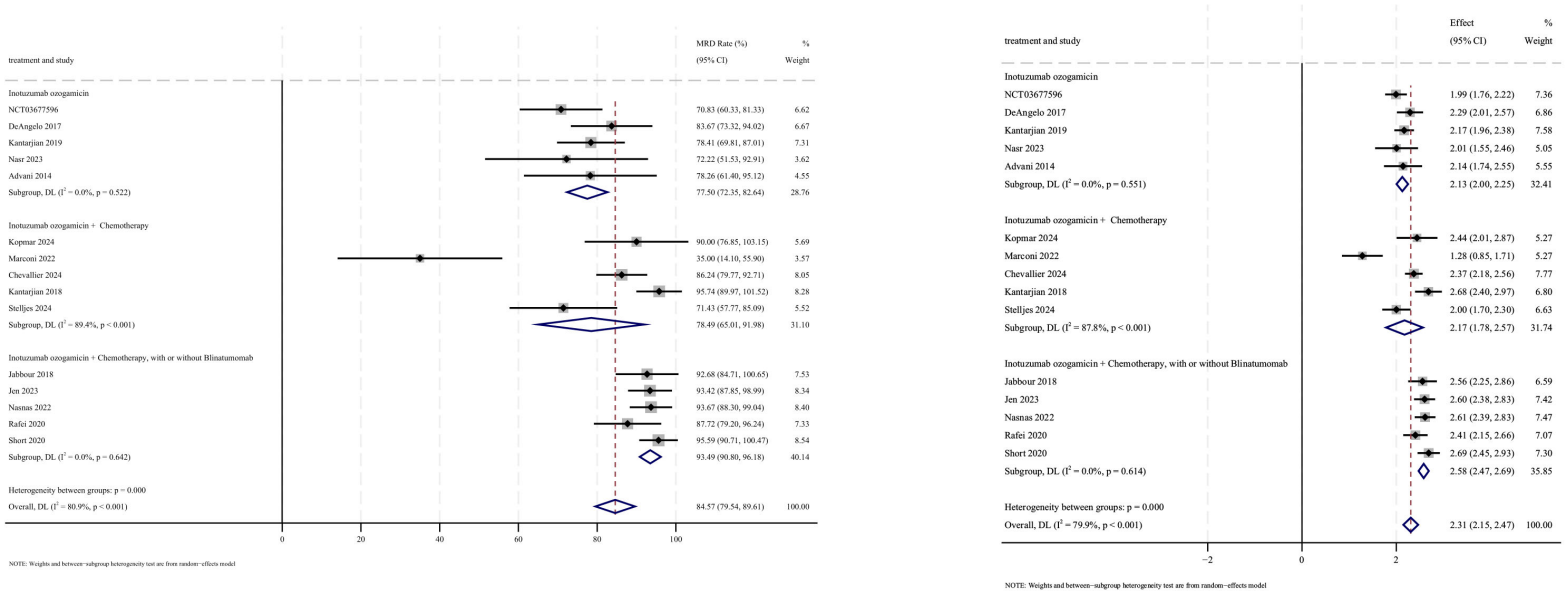
Forest map for subgroup analysis of treatment regimens in MRD.

**Figure 7:**
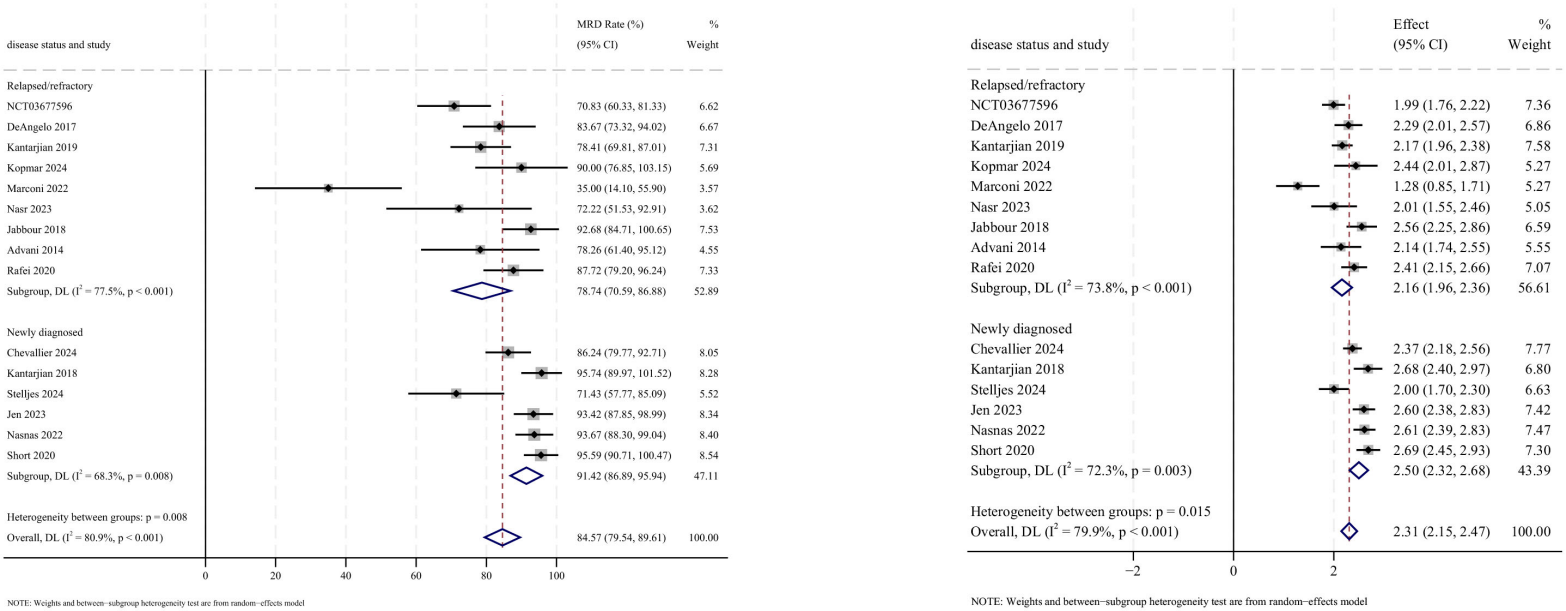
Forest map for subgroup analysis of disease status in MRD.

MRD− outcomes were reported in 15 studies, with analysis using a random-effects model yielding a pooled MRD− rate of 84.6% (95% CI: 79.5%–89.6%, 95% PI: 0.4%–99.8%; *I* ^2^ = 80.9%, *p*<0.001) (**Figures 6**, **7**). Subgroup analyses revealed two findings: (1) InO combined with chemotherapy, with or without blinatumomab demonstrated the highest MRD− rate at 93.5% (95% CI: 90.8%–96.2%; *I* ^2^ = 0.0%, *p* =0.642); (2) newly diagnosed patients with ALL showed a superior MRD− rate of 91.4% (95% CI: 86.9%–95.9%; *I* ^2^ = 68.3%, *p* =0.008). Sensitivity analysis employing the leave-one-out method confirmed that all effect sizes remained within the 95% CIs, indicating the robust stability of the findings (**Supplementary Material 2**). Both the funnel plot (**Supplementary Material 3**) and Egger’s linear regression test (*p* =0.179, **Supplementary Material 4**) showed no significant evidence of publication bias.

### 3.4 Survival

#### 3.4.1 Overall survival for 1 year

The 1-year OS rate was directly obtained from the numerical reports of eight included studies (19–21, 23–25, 27, 28). The pooled 1-year OS rate was 61.7% (95% CI: 44.9%–78.5%; *I* ^2^ = 94.1%, *p*<0.001) (**Supplementary Material 5**). Subgroup analysis revealed that newly diagnosed patients with ALL demonstrated significantly superior survival outcomes of 82.7% (95% CI: 71.2%–94.2%; *I* ^2^ = 78.1%, *p* =0.010) compared to R/R cases.

#### 3.4.2 Overall survival for 2 years

The 2-year OS rate was directly obtained from the numerical reports of five included studies (18, 21–23, 26). The pooled 2-year OS rate was 51.4% (95% CI: 32.2%–70.7%; *I* ^2^ = 93.8%, *p*<0.001) (**Supplementary Material 5**). Subgroup analysis revealed that newly diagnosed patients with ALL exhibited significantly better survival outcomes of 59.0% (95% CI: 49.0%–68.9%; *I* ^2^ = 42.5%, *p* =0.187) compared to R/R cases.

#### 3.4.3 Overall survival for 3 years

The 3-year OS rate was directly obtained from the numerical reports of four included studies (18, 22, 24, 31). The pooled 3-year OS rate was 46.9% (95% CI: 22.5%–71.4%, 95%; *I* ^2^ = 95.2%, *p*<0.001) (**Supplementary Material 5**). Subgroup analysis revealed that newly diagnosed patients with ALL achieved significantly superior 3-year survival rates of 64.0% (95% CI: 48.0%–80.0%; *I* ^2^ = 64.6%, *p* =0.093) compared to R/R cases.

#### 3.4.4 Overall survival for 5 years

The 5-year OS rate was directly obtained from the numerical reports of three included studies (21, 29, 30). The pooled 5-year OS rate was 44.9% (95% CI: 39.2%–50.5%, 95%; *I* ^2^ = 0.0%, *p* =0.482) (**Supplementary Material 5**).

### 3.5 Stem cell transplantation

**Figure 8:**
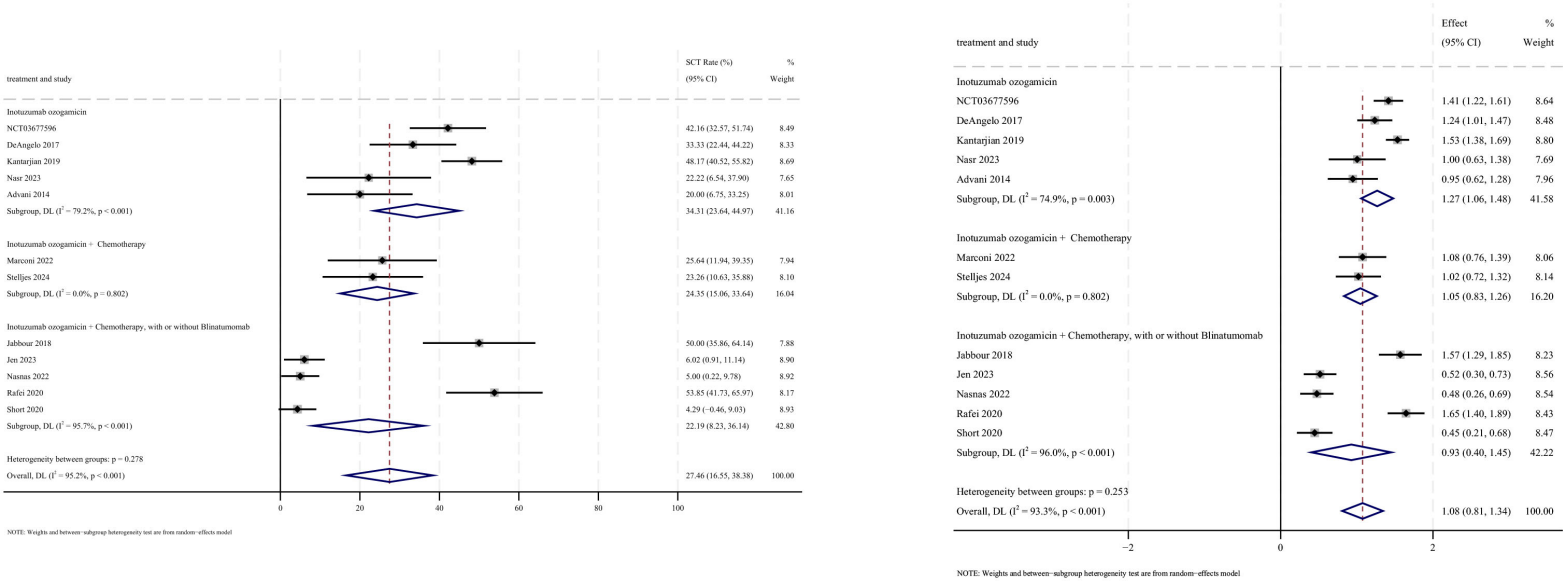
Forest map for subgroup analysis of treatment regimens in SCT.

**Figure 9:**
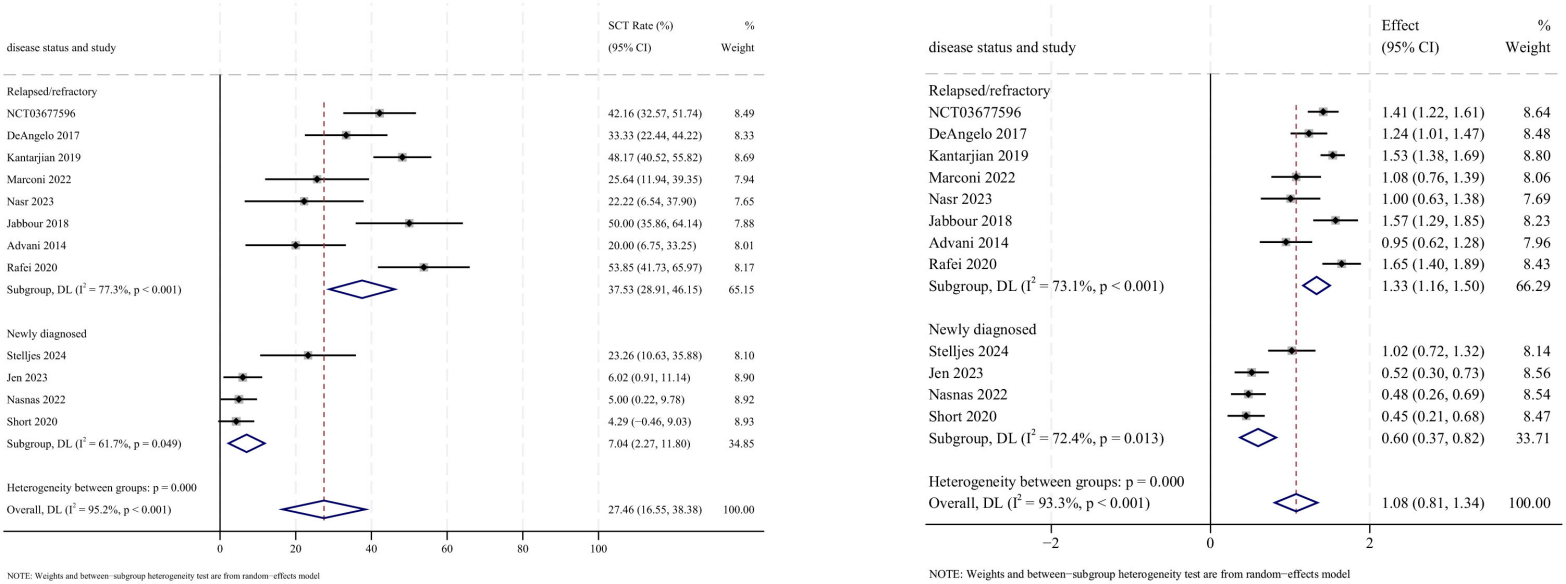
Forest map for subgroup analysis of disease status in SCT.

Allogeneic stem cell transplantation (allo-SCT) was reported as the subsequent treatment following InO therapy in all 12 studies that provided data on transplantation rates. The pooled SCT rate was 27.5% (95% CI: 16.6%–38.4%, 95% PI: 1.2%–79.4%; *I* ^2^ = 95.2%, *p*<0.001) (**Figures 8**, **9**). Subgroup analyses revealed two findings: (1) InO monotherapy demonstrated the highest SCT rate at 34.3% (95% CI: 23.6%–45.0%; *I* ^2^ = 79.2%, *p*<0.001); (2) patients with R/R ALL showed a superior SCT rate of 37.5% (95% CI: 28.9%–46.1%; *I* ^2^ = 77.3%, *p*<0.001). Sensitivity analysis using the leave-one-out method confirmed result stability (**Supplementary Material 2**). Both the funnel plot (**Supplementary Material 3**) and Egger’s linear regression test (*p* =0.444, **Supplementary Material 4**) showed no significant evidence of publication bias.

### 3.6 Relapse

**Figure 10:**
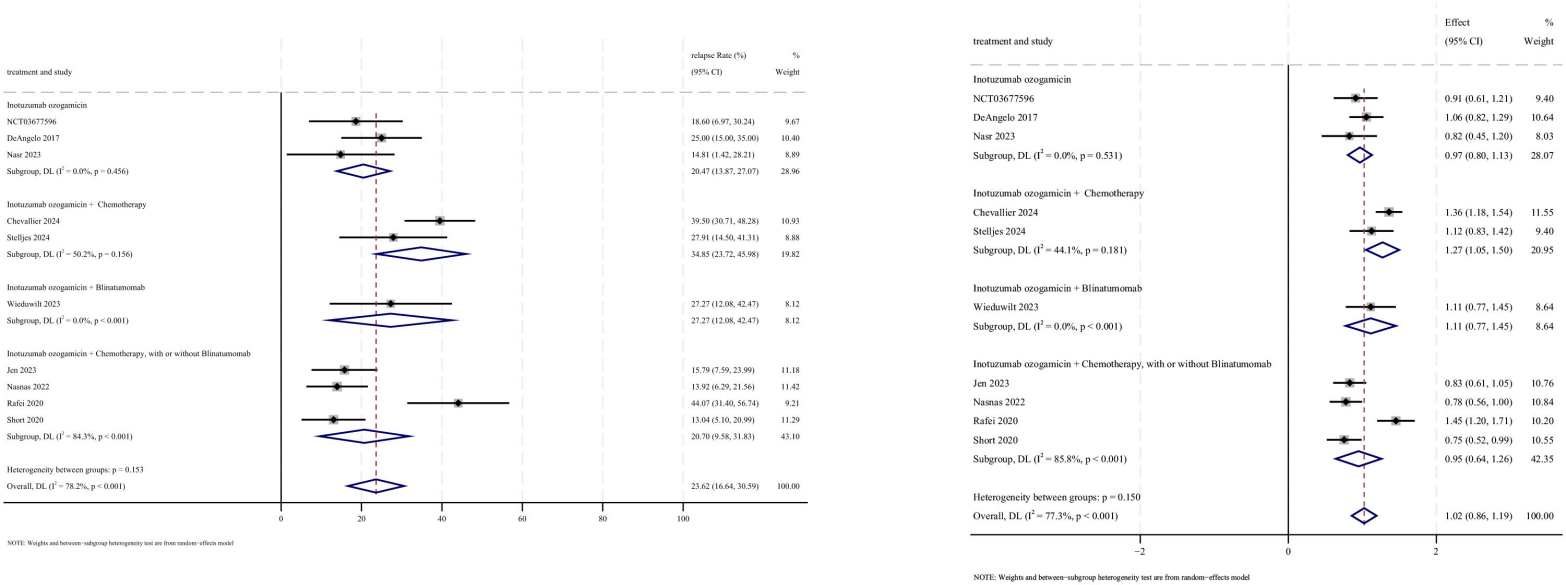
Forest map for subgroup analysis of treatment regimens in relapse.

**Figure 11:**
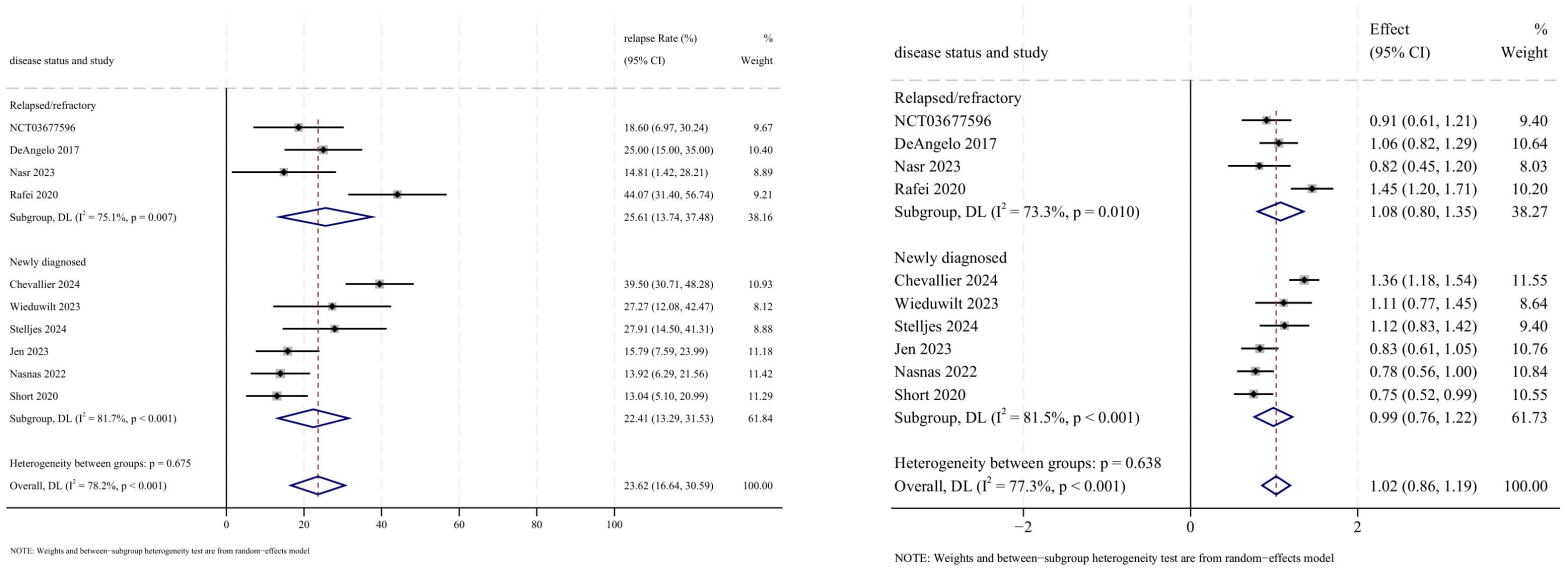
Forest map for subgroup analysis of disease status in relapse.

Relapse outcomes were reported in 10 studies, with analysis using a random-effects model yielding a pooled relapse rate of 23.6% (95% CI: 16.6%–30.6%, 95% PI: 16.6%–99.6%; *I* ^2^ = 78.2%, *p*<0.001) (**Figures 10**, **11**). Subgroup analyses revealed two findings: (1) InO monotherapy regimen demonstrated the lowest relapse rate at 20.5% (95% CI: 13.9%–27.0%; *I* ^2^ = 0.0%, *p* =0.456); (2) newly diagnosed patients with ALL had a lower incidence of relapse rate at 22.4% (95% CI: 13.3%–31.5%; *I* ^2^ = 81.7%, *p*<0.001). Sensitivity analysis using the leave-one-out approach confirmed result stability (**Supplementary Material 2**). Both the funnel plot (**Supplementary Material 3**) and Egger’s linear regression test (*p* =0.511, **Supplementary Material 4**) showed no significant evidence of publication bias.

### 3.7 Safety

**Figure 12:**
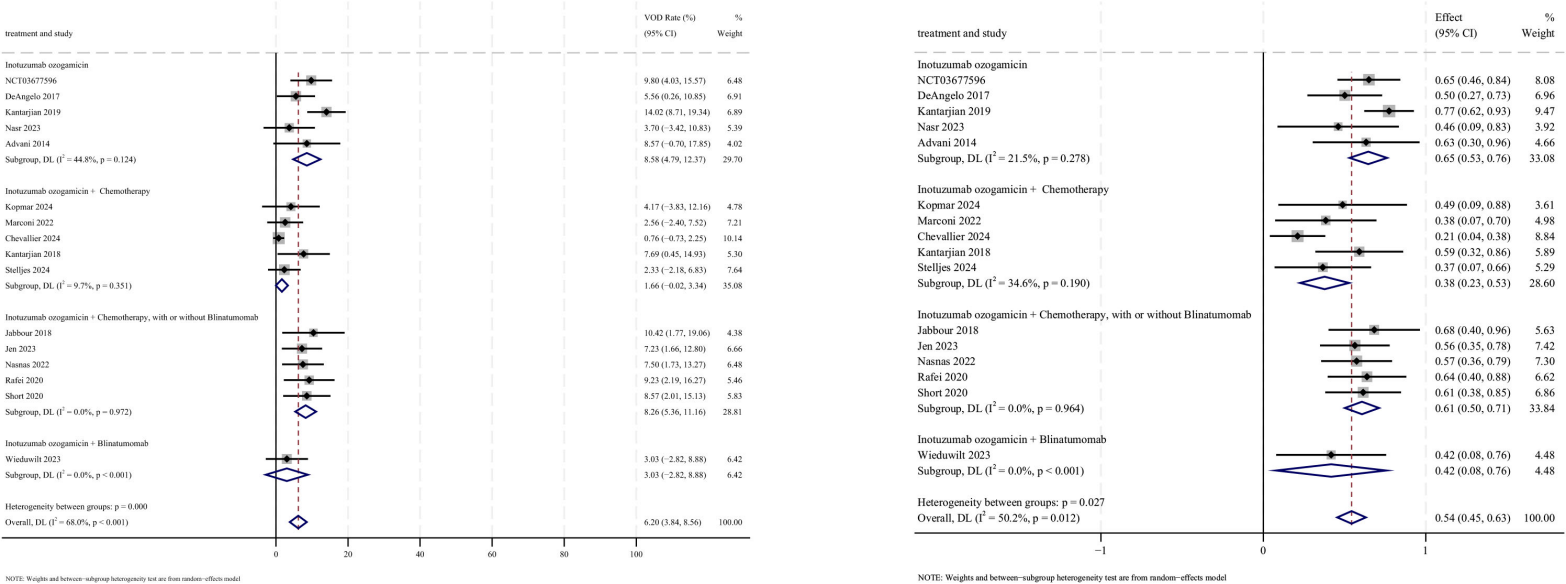
Forest map for subgroup analysis of treatment regimens in VOD.

**Figure 13:**
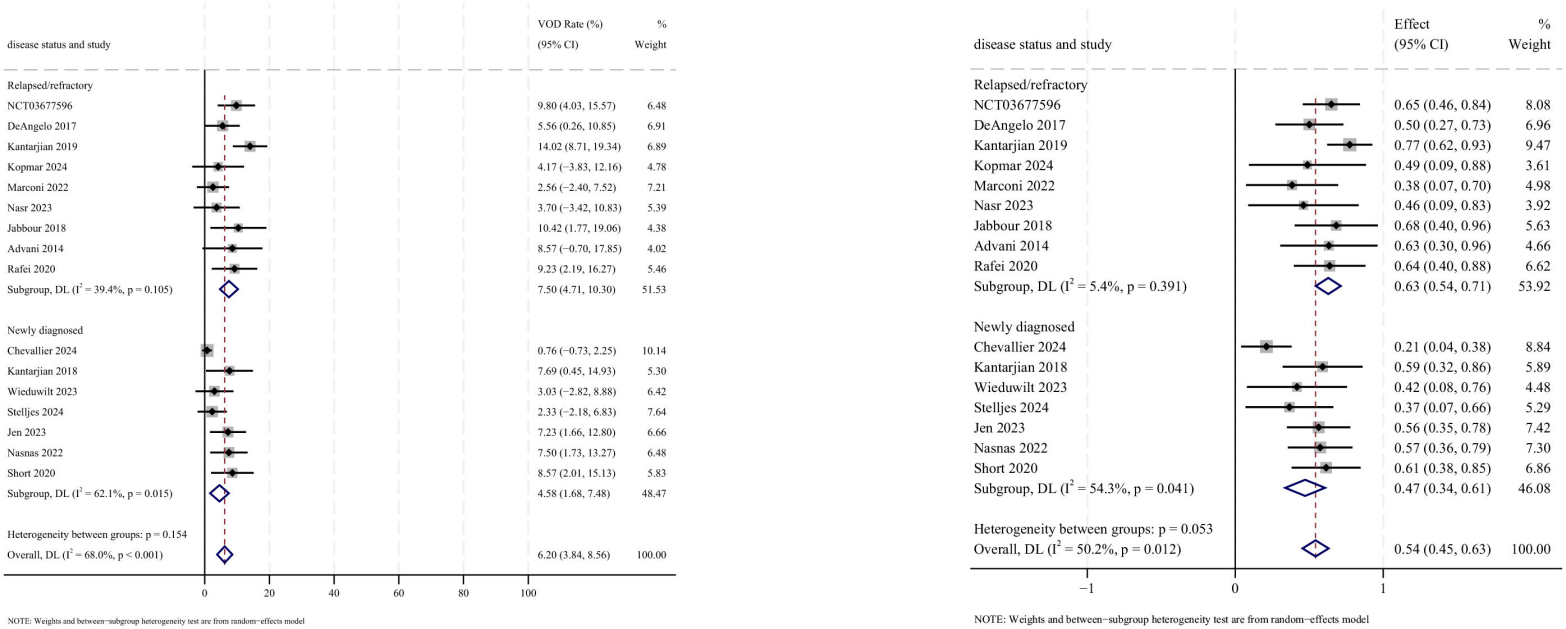
Forest map for subgroup analysis of disease status in VOD.

AEs were reported in 16 studies, all of which documented the incidence of veno-occlusive disease (VOD) or sinusoidal obstruction syndrome (SOS). The pooled incidence of VOD/SOS was 6.2% (95% CI: 3.8%–8.6%, 95% PI: 6.6%–54.5%; *I* ^2^ = 68.0%, *p*<0.001) (**Figures 12**, **13**). Subgroup analyses revealed the following findings: (1) InO combined with chemotherapy demonstrated the lowest incidence of VOD/SOS at 1.7% (95% CI: −0.02%–3.3%; *I* ^2^ = 9.7%, *p* =0.351); (2) newly diagnosed patients with ALL had a lower incidence of VOD/SOS at 4.6% (95% CI: 1.7%–7.5%; *I* ^2^=62.1%, *p* =0.015). Sensitivity analysis using the leave-one-out method confirmed result stability (**Supplementary Material 2**). Both the funnel plot (**Supplementary Material 3**) and Egger’s linear regression test (*p* =0.517, **Supplementary Material 4**) showed no significant evidence of publication bias. Other commonly reported AEs included thrombocytopenia, neutropenia, anemia, fatigue, nausea, vomiting, and hyperbilirubinemia, as detailed in **Table 2** (18–24, 28).

**Table 2:** Pooled results of common AEs of any grade and ≥grade 3.

AEs	Any grade		≥Grade 3	
	ES, % (95% CI)	*I* ^2^, %	ES, % (95% CI)	*I* ^2^, %
Thrombocytopenia (19, 24, 28)	45.8 (22.7–66.8)	88.9	37.4 (27.2–47.5)	40.3
Neutropenia (19, 24, 28)	36.9 (17.6–56.1)	84.5	35.1 (16.3–53.8)	84.0
Febrile neutropenia (18–20)	16.4 (9.7–23.2)	48.7	11.0 (4.0–18.0)	74.1
Anemia (23, 24)	83.4 (53.6–113.3)	94.0	60.5 (15.3–105.7)	96.1
Hyperbilirubinemia (22–24)	49.9 (5.6–105.5)	99.0	14.7 (7.8–21.6)	0.0
Nausea (19, 22, 23)	49.4 (18.3–80.5)	95.1	2.0 (1.0–4.0)	13.8
Vomiting (19, 22, 23)	22.5 (4.3–30.7)	41.3	4.0 (1.0–8.0)	65.9
Fatigue (19, 22, 23)	41.3 (11.8–70.7)	94.6	2.0 (1.0–5.0)	38.1

## 4 Discussion

This systematic review and meta-analysis provides a comprehensive evaluation of the efficacy and safety of InO in the treatment of adult ALL. By analyzing 16 clinical studies, including 1,068 patients, several key findings emerged that have important clinical implications. First, InO regimens showed robust efficacy: combination therapies surpassed monotherapy in clinical outcomes and safety, while newly diagnosed patients responded better than R/R cases. Second, InO showed significant short- and long-term survival benefits for patients with ALL. Third, VOD and SOS were identified as characteristic toxicities, though the incidence remained low across studies. Importantly, these findings support InO’s potential as a viable therapeutic option for adult patients with ALL, particularly those with R/R disease.

Since the last meta-analysis (12), multiple new studies (20, 21, 27, 29, 30) have evaluated InO-based combination therapies, providing more robust data. While a direct quantitative comparison with other immunotherapies is constrained by inter-study heterogeneity, our results for InO can be qualitatively contextualized alongside published pooled estimates for blinatumomab and CAR-T therapy (33). In terms of efficacy, InO’s performance in CR rates, MRD negativity, and OS appears intermediary relative to these other strategies. Regarding safety, the profiles are distinct: blinatumomab and CAR-T therapy are predominantly associated with cytokine release syndrome and neurotoxicity, whereas InO carries a significant risk of VOD. Overall, this analysis positions InO as a modality with intermediate efficacy and a unique, clinically manageable toxicity profile within the R/R ALL treatment sequence.

InO-based combination regimens demonstrated superior efficacy compared with monotherapy, as reflected by higher OR and CR rates. The enhanced therapeutic effect likely arises from complementary cytotoxic mechanisms targeting distinct antigens. InO delivers the cytotoxic agent calicheamicin into leukemic cells via CD22-mediated endocytosis, inducing DNA double-strand breaks, whereas blinatumomab redirects patient-derived T cells to CD19+ leukemic cells, promoting cytolytic activity. Together, these agents target more than 90% of B-ALL blasts and mitigate the risk of monoantigen escape (23). This dual-targeting strategy provides reciprocal antigen coverage: clones with low or resistant CD22 expression can be eradicated by CD19-directed blinatumomab, and vice versa, forming a “double-insurance” mechanism. Moreover, combining InO or blinatumomab with low-intensity chemotherapy regimens such as mini-hyper-CVD reduces overall treatment intensity and toxicity, thereby maintaining high remission rates while minimizing the hematologic and organ-related adverse effects associated with conventional high-dose chemotherapy (34). Although combination therapy achieved superior remission rates compared with monotherapy, it was paradoxically associated with a higher relapse rate. This discrepancy likely reflects baseline imbalances between treatment cohorts. In clinical practice, combination regimens are preferentially administered to patients with newly diagnosed or high-risk disease, greater tumor burden, or more aggressive biological subtypes. Consequently, the combination therapy group may have included patients with inherently higher relapse risk at baseline, contributing to the observed difference (22). By contrast, the principal therapeutic goal for R/R patients is often to achieve remission sufficient for HSCT. Despite having resistant disease, these patients are typically selected based on stable clinical status and preserved organ function that allows transplantation. InO monotherapy thus serves as an effective bridge-to-transplant strategy. The fact that the monotherapy subgroup in this meta-analysis consisted exclusively of R/R patients supports this interpretation; accordingly, the lower relapse rate observed in this cohort is consistent with expected clinical outcomes.

Newly diagnosed patients with ALL exhibited superior treatment responses, which likely reflect differences in disease biology, immune competence, and baseline clinical status compared with R/R patients. In contrast, individuals with R/R ALL demonstrated poorer outcomes, underscoring the inherent therapeutic challenges of advanced disease, where multidrug resistance and profound immune dysfunction often limit treatment efficacy. These findings emphasize the critical importance of early detection and timely intervention, highlighting the need to optimize frontline therapeutic strategies (35). While InO demonstrated strong efficacy in Philadelphia chromosome-negative (Ph−) ALL, responses were comparatively inferior in Philadelphia chromosome-positive (Ph+) cases (25, 26). This discrepancy may be attributable to the greater genetic heterogeneity and aggressive disease biology of Ph+ ALL (36), as well as the lower CD22 antigen density on Ph+ blasts (37), which may impair InO binding and intracellular delivery. Kantarjian et al. (38) further reported that higher CD22 expression correlates with improved responsiveness to InO, suggesting that enhancing CD22 targeting could potentiate therapeutic benefit. Future studies might explore combination approaches with other targeted agents or interventions capable of upregulating CD22 expression.

In the majority of studies included in this meta-analysis, the proportion of enrolled patients with prior InO exposure was not specifically reported. This raises a critical question for clinical practice: how to treat patients who have already received InO and may have developed resistance. Resistance to InO is often associated with diminished CD22 surface expression or impaired internalization, potentially leading to treatment failure upon re-challenge (39). In such cases, leveraging alternative immunotherapies that target non-overlapping antigens, such as CD19-directed bispecific T-cell engagers (e.g., blinatumomab) or CAR T-cell therapy, represents a rational therapeutic strategy. This sequential approach—using agents with complementary mechanisms of action—could help circumvent the issue of antigen escape and serve as a “dual insurance” in the treatment continuum. Future studies are urgently needed to validate optimal sequencing strategies and to define effective therapeutic options for patients with prior InO exposure.

Despite InO’s promising efficacy, the occurrence of VOD/SOS remains a concern. The incidence of VOD/SOS was higher in patients receiving InO monotherapy, particularly in R/R cases. However, the incidence was lower in newly diagnosed patients, possibly reflecting differences in baseline liver function and overall health. Notably, our study may have underestimated the VOD/SOS risk. As a bridge to HSCT, InO represents an established risk factor for drug-induced liver injury and SOS/VOD. Its intrinsic VOD/SOS risk may synergize with transplantation-related toxicity, potentially exacerbating the incidence of VOD/SOS in patients proceeding to HSCT (40). In clinical practice, the risk of VOD/SOS can be mitigated through vigilant monitoring, including baseline liver function assessments and the use of prophylactic measures such as ursodeoxycholic acid. Additionally, institutions administering InO should consider implementing hepatic fibrosis screening (e.g., portal vein Doppler ultrasound) to reduce the risk of VOD/SOS, particularly in patients undergoing HSCT (41). Interestingly, we observed a paradoxical clinical pattern: while CR/MRD− patients represent optimal transplant candidates, the monotherapy group (all R/R ALL) demonstrated higher SCT rates than combination cohorts. This likely reflects therapeutic decision-making favoring salvage SCT in refractory cases, even with suboptimal responses (42).

Our study has several limitations. First, the majority of included studies were single-arm trials, limiting direct comparison of InO’s efficacy with other treatments. Consequently, comparisons were made with historical controls, which may introduce significant heterogeneity. Second, the observed heterogeneity in treatment regimens, patient demographics, and disease characteristics could potentially impact the stability of our results. Lastly, a substantial proportion of the included studies were conference abstracts, which often lack detailed information, thereby limiting thorough assessment of study quality and introducing potential bias.

## 5 Conclusion

In summary, our pooled analysis indicates that InO-based therapies show promising efficacy and manageable safety in ALL treatment, with infrequent occurrences of severe VOD or grade ≥3 AEs. Additional high-quality RCTs are needed to confirm the therapeutic potential of InO and to better define its therapeutic position in ALL management.

## Data Availability

The original contributions presented in the study are included in the article/**Supplementary Material**. Further inquiries can be directed to the corresponding author.
