# Supplementary material for: Efficacy and safety of inotuzumab ozogamicin and its combination therapies in acute lymphoblastic leukemia: a systematic review and meta-analysis
Source: Front Oncol. 2025 Nov 4;15:1613777. doi: 10.3389/fonc.2025.1613777 (PMC12623164; doi:10.3389/fonc.2025.1613777)
Supplement: Supplementary file 1 [file DataSheet1.docx]

Supplementary Material

# Search result record

| The search strategy of PubMed | | | | |
| --- | --- | --- | --- | --- |
| # | Query | | | Results |
| #1 | Precursor Cell Lymphoblastic Leukemia Lymphoma[MeSH Terms] | | | 35238 |
| #2 | acute lymphatic leukaemia[Title/Abstract] OR acute lymphatic leukemia[Title/Abstract] OR acute lymphoblastic leukaemia[Title/Abstract] OR acute lymphoblastic leukemia[Title/Abstract] OR acute lymphoblastic leukemia lymphoma[Title/Abstract] OR acute lymphoblastic leukemias acute lymphocyte leukaemia[Title/Abstract] OR acute lymphocyte leukemia[Title/Abstract] OR acute lymphocytic leukaemia[Title/Abstract] OR acute lymphocytic leukemia[Title/Abstract] OR acute lymphoid leukaemia[Title/Abstract] OR acute lymphoid leukemia[Title/Abstract] OR CALLA positive leukaemia[Title/Abstract] OR CALLA positive leukemia[Title/Abstract] OR Childhood ALL[Title/Abstract] OR L1 acute lymphocytic leukaemia[Title/Abstract] OR L1 acute lymphocytic leukemia[Title/Abstract] OR L1 Lymphocytic Leukemia[Title/Abstract] OR L2 acute lymphocytic leukaemia[Title/Abstract] OR L2 acute lymphocytic leukemia[Title/Abstract] OR L2 Lymphocytic Leukemia[Title/Abstract] OR Lymphoblastic Leukemia[Title/Abstract] OR Lymphoblastic Lymphoma[Title/Abstract] OR null cell leukaemia[Title/Abstract] OR null cell leukemia[Title/Abstract] | | | 49271 |
| #3 | Inotuzumab ozogamicin[MeSH Terms] | | | 262 |
| #4 | Inotuzumab ozogamicin[Title/Abstract] OR CMC-544[Title/Abstract] OR Besponsa[Title/Abstract] | | | 393 |
| #5 | (#1 OR #2) AND (#3 OR #4) | | | 332 |
| The search strategy of Embase | | | | |
| # | | Query | Results | |
| #1 | | 'acute lymphoblastic leukemia'/exp | 106436 | |
| #2 | | 'acute lymphatic leukaemia':ab,ti,kw OR 'acute lymphatic leukemia':ab,ti,kw OR 'acute lymphoblastic leukaemia':ab,ti,kw OR 'acute lymphoblastic leukemia':ab,ti,kw OR 'acute lymphoblastic leukemia lymphoma':ab,ti,kw OR 'acute lymphoblastic leukemias':ab,ti,kw OR 'acute lymphocyte leukaemia':ab,ti,kw OR 'acute lymphocyte leukemia':ab,ti,kw OR 'acute lymphocytic leukaemia':ab,ti,kw OR 'acute lymphocytic leukemia':ab,ti,kw OR 'acute lymphoid leukaemia':ab,ti,kw OR 'acute lymphoid leukemia':ab,ti,kw OR 'CALLA positive leukaemia':ab,ti,kw OR 'CALLA positive leukemia':ab,ti,kw OR 'Childhood ALL':ab,ti,kw OR 'L1 acute lymphocytic leukaemia':ab,ti,kw OR 'L1 acute lymphocytic leukemia':ab,ti,kw OR 'L1 Lymphocytic Leukemia':ab,ti,kw OR 'L2 acute lymphocytic leukaemia':ab,ti,kw OR 'L2 acute lymphocytic leukemia':ab,ti,kw OR 'L2 Lymphocytic Leukemia':ab,ti,kw OR 'Lymphoblastic Leukemia':ab,ti,kw OR 'Lymphoblastic Lymphoma':ab,ti,kw OR 'null cell leukaemia':ab,ti,kw OR 'null cell leukemia':ab,ti,kw OR 'Precursor Cell Lymphoblastic Leukemia Lymphoma':ab,ti,kw | 75611 | |
| #3 | | 'Inotuzumab ozogamicin'/exp | 2144 | |
| #4 | | 'Inotuzumab ozogamicin':ab,ti,kw OR 'CMC-544':ab,ti,kw OR 'Besponsa':ab,ti,kw | 72 | |
| #5 | | (#1 OR #2) AND (#3 OR #4) | 1464 | |

| The search strategy of Cochrane Library | | |
| --- | --- | --- |
| # | Query | Results |
| #1 | 'Precursor Cell Lymphoblastic Leukemia Lymphoma'/exp | 1590 |
| #2 | 'acute lymphatic leukaemia':ab,ti,kw OR 'acute lymphatic leukemia':ab,ti,kw OR 'acute lymphoblastic leukaemia':ab,ti,kw OR 'acute lymphoblastic leukemia':ab,ti,kw OR 'acute lymphoblastic leukemia lymphoma':ab,ti,kw OR 'acute lymphoblastic leukemias':ab,ti,kw OR 'acute lymphocyte leukaemia':ab,ti,kw OR 'acute lymphocyte leukemia':ab,ti,kw OR 'acute lymphocytic leukaemia':ab,ti,kw OR 'acute lymphocytic leukemia':ab,ti,kw OR 'acute lymphoid leukaemia':ab,ti,kw OR 'acute lymphoid leukemia':ab,ti,kw OR 'CALLA positive leukaemia':ab,ti,kw OR 'CALLA positive leukemia':ab,ti,kw OR 'Childhood ALL':ab,ti,kw OR 'L1 acute lymphocytic leukaemia':ab,ti,kw OR 'L1 acute lymphocytic leukemia':ab,ti,kw OR 'L1 Lymphocytic Leukemia':ab,ti,kw OR 'L2 acute lymphocytic leukaemia':ab,ti,kw OR 'L2 acute lymphocytic leukemia':ab,ti,kw OR 'L2 Lymphocytic Leukemia':ab,ti,kw OR 'Lymphoblastic Leukemia':ab,ti,kw OR 'Lymphoblastic Lymphoma':ab,ti,kw OR 'null cell leukaemia':ab,ti,kw OR 'null cell leukemia':ab,ti,kw OR 'Precursor Cell Lymphoblastic Leukemia Lymphoma':ab,ti,kw | 10580 |
| #3 | 'Inotuzumab ozogamicin'/exp | 34 |
| #4 | 'Inotuzumab ozogamicin':ab,ti,kw OR 'CMC-544':ab,ti,kw OR 'Besponsa':ab,ti,kw | 6 |
| #5 | (#1 OR #2) AND (#3 OR #4) | 35 |

| The search strategy of Web of Science | |
| --- | --- |
| Query | Results |
| #1:TS=('acute lymphatic leukaemia' OR 'acute lymphatic leukemia' OR 'acute lymphoblastic leukaemia' OR 'acute lymphoblastic leukemia' OR 'acute lymphoblastic leukemia lymphoma' OR 'acute lymphoblastic leukemias' OR 'acute lymphocyte leukaemia' OR 'acute lymphocyte leukemia' OR 'acute lymphocytic leukaemia' OR 'acute lymphocytic leukemia' OR 'acute lymphoid leukaemia' OR 'acute lymphoid leukemia' OR 'CALLA positive leukaemia' OR 'CALLA positive leukemia' OR 'Childhood ALL' OR 'L1 acute lymphocytic leukaemia' OR 'L1 acute lymphocytic leukemia' OR 'L1 Lymphocytic Leukemia' OR 'L2 acute lymphocytic leukaemia' OR 'L2 acute lymphocytic leukemia' OR 'L2 Lymphocytic Leukemia' OR 'Lymphoblastic Leukemia' OR 'Lymphoblastic Lymphoma' OR 'null cell leukaemia' OR 'null cell leukemia' OR 'Precursor Cell Lymphoblastic Leukemia Lymphoma') | 176136 |
| #2:TS=('Inotuzumab ozogamicin' OR 'CMC-544' OR 'Besponsa') | 920 |
| #3:#1 AND #2 | 672 |

| The search strategy of CT.gov | |
| --- | --- |
| Query | Results |
| （acute lymphoblastic leukemia OR ALL）AND（Inotuzumab ozogamicin OR CMC-544） | 65 |
