# Supplementary material for: Efficacy and safety of inotuzumab ozogamicin and its combination therapies in acute lymphoblastic leukemia: a systematic review and meta-analysis
Source: Front Oncol. 2025 Nov 4;15:1613777. doi: 10.3389/fonc.2025.1613777 (PMC12623164; doi:10.3389/fonc.2025.1613777)

Supplementary Material

# Figure 1 Sensitivity analysis of OR. OR, over response.


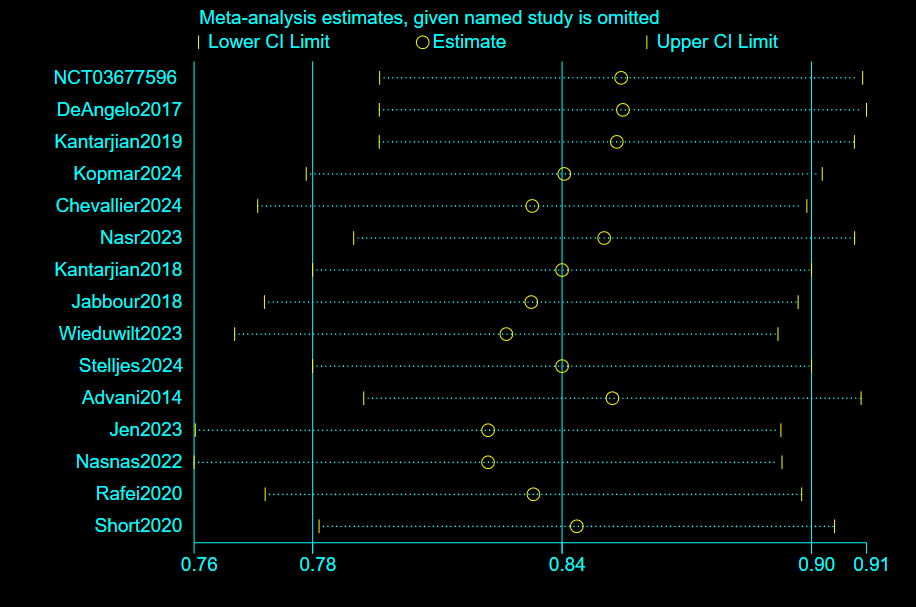


# Figure 2 Sensitivity analysis of CR. CR, complete remission.


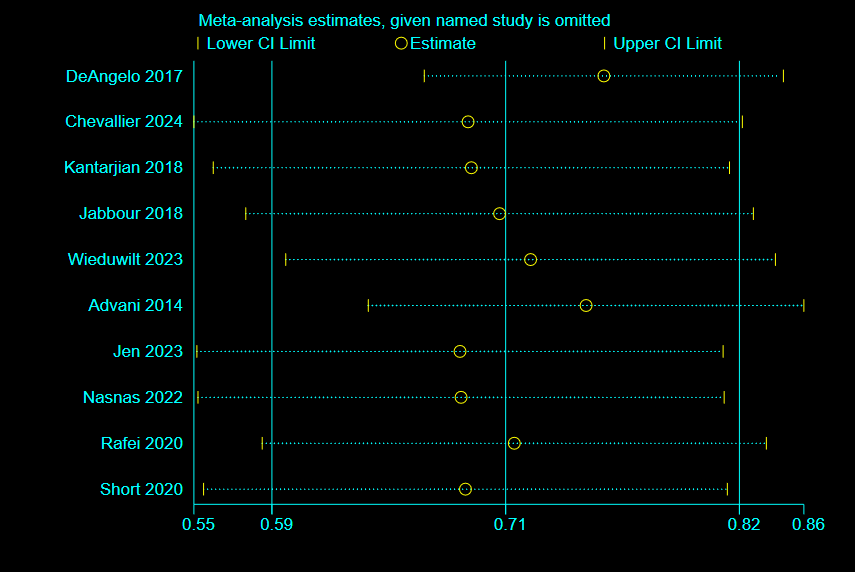


# Figure 3 Sensitivity analysis of MRD. MRD, minimal residual disease.


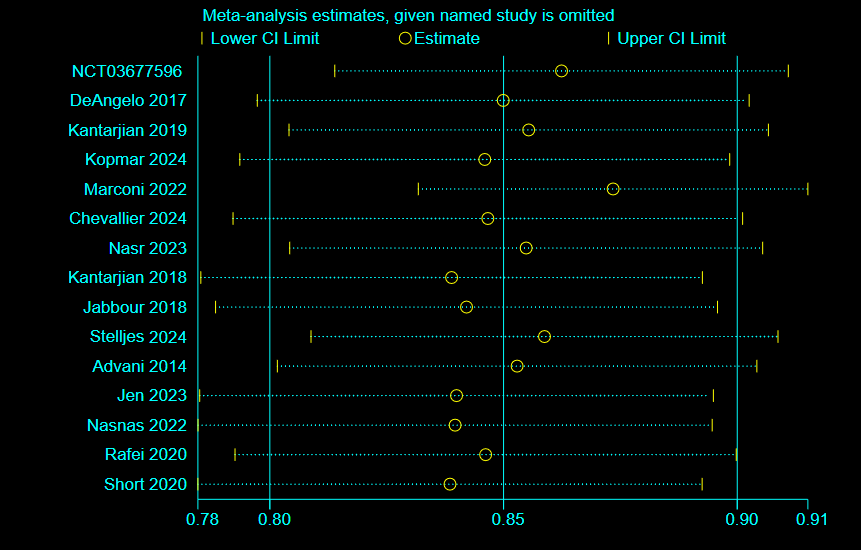


# Figure 4 Sensitivity analysis of SCT.SCT, stem cell transplantation.


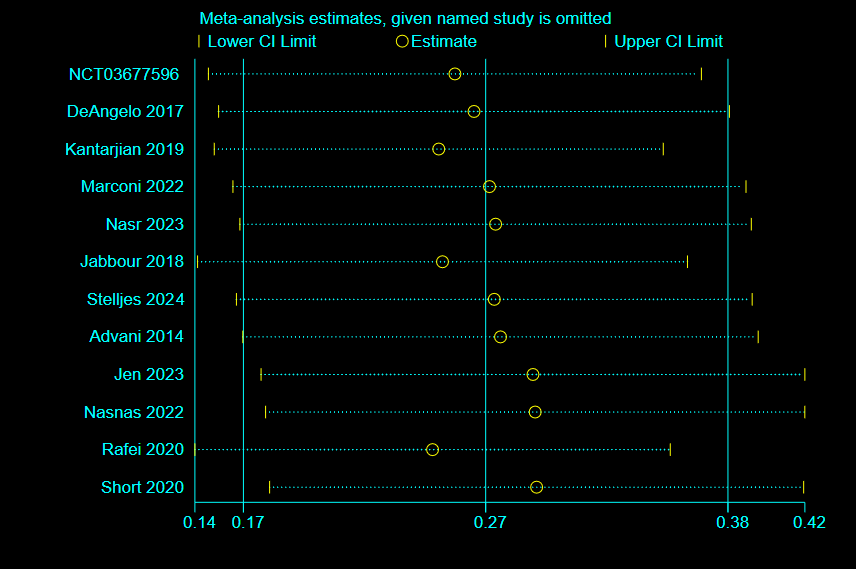


# Figure 5 Sensitivity analysis of VOD. VOD, venous occlusive disease.


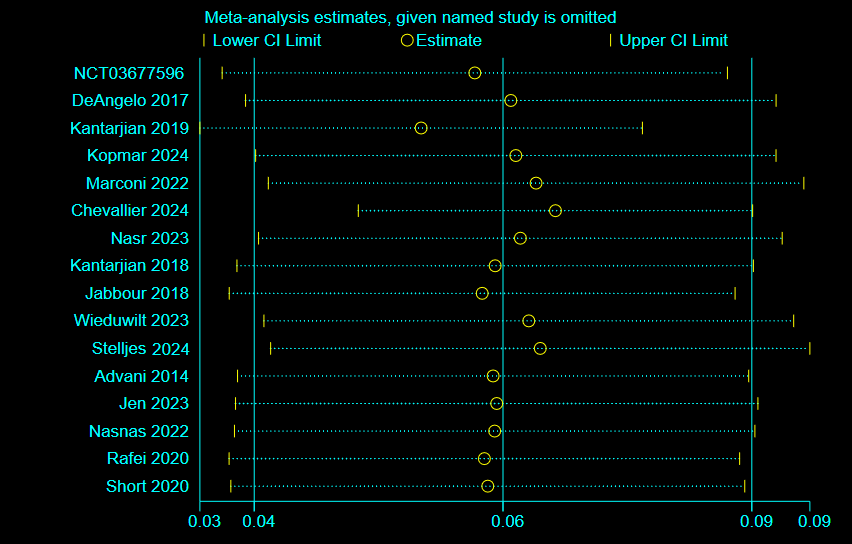


# Figure 6 Sensitivity analysis of relapse.


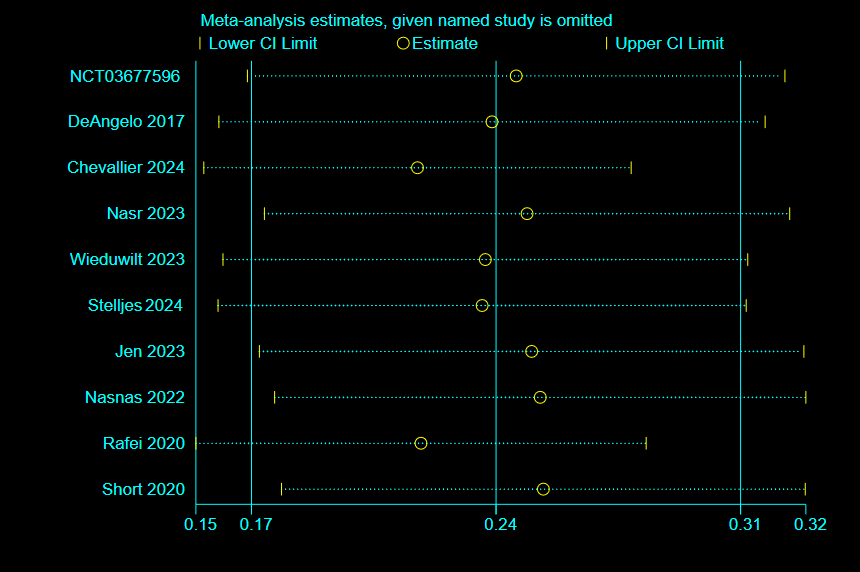

Supplement: Supplementary file 2 [file DataSheet2.docx]
