# Supplementary material for: Efficacy and safety of inotuzumab ozogamicin and its combination therapies in acute lymphoblastic leukemia: a systematic review and meta-analysis
Source: Front Oncol. 2025 Nov 4;15:1613777. doi: 10.3389/fonc.2025.1613777 (PMC12623164; doi:10.3389/fonc.2025.1613777)

Supplementary Material

# Figure 1 Forest map for subgroup analysis of disease status in OS（1 year）

#
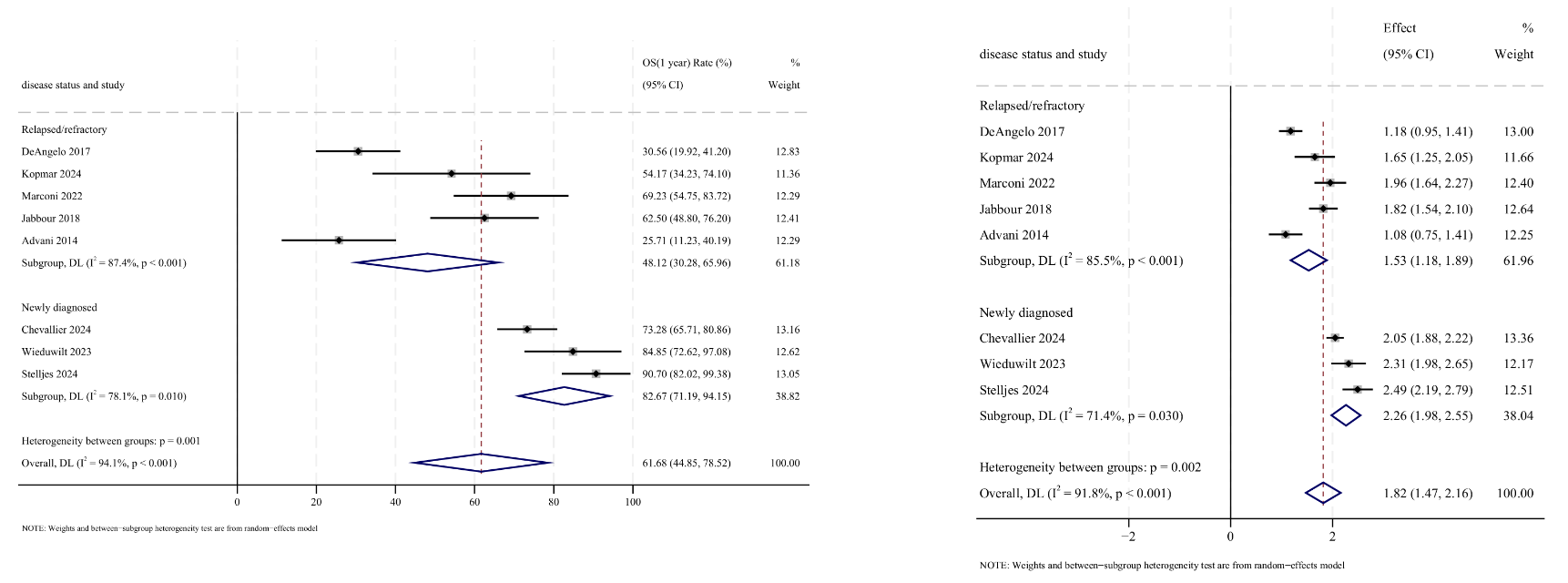


# Figure 2 Forest map for subgroup analysis of disease status in OS（2 years）

#
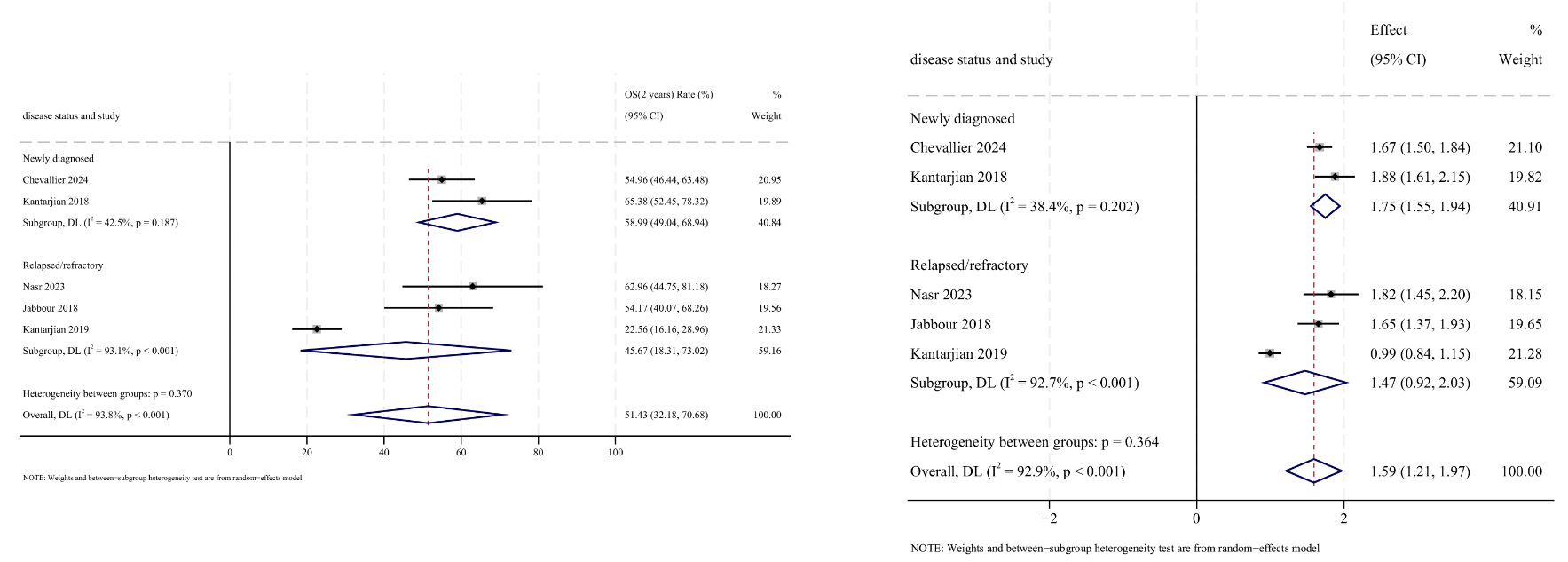


# Figure 3 Forest map for subgroup analysis of disease status in OS（3 years）

#
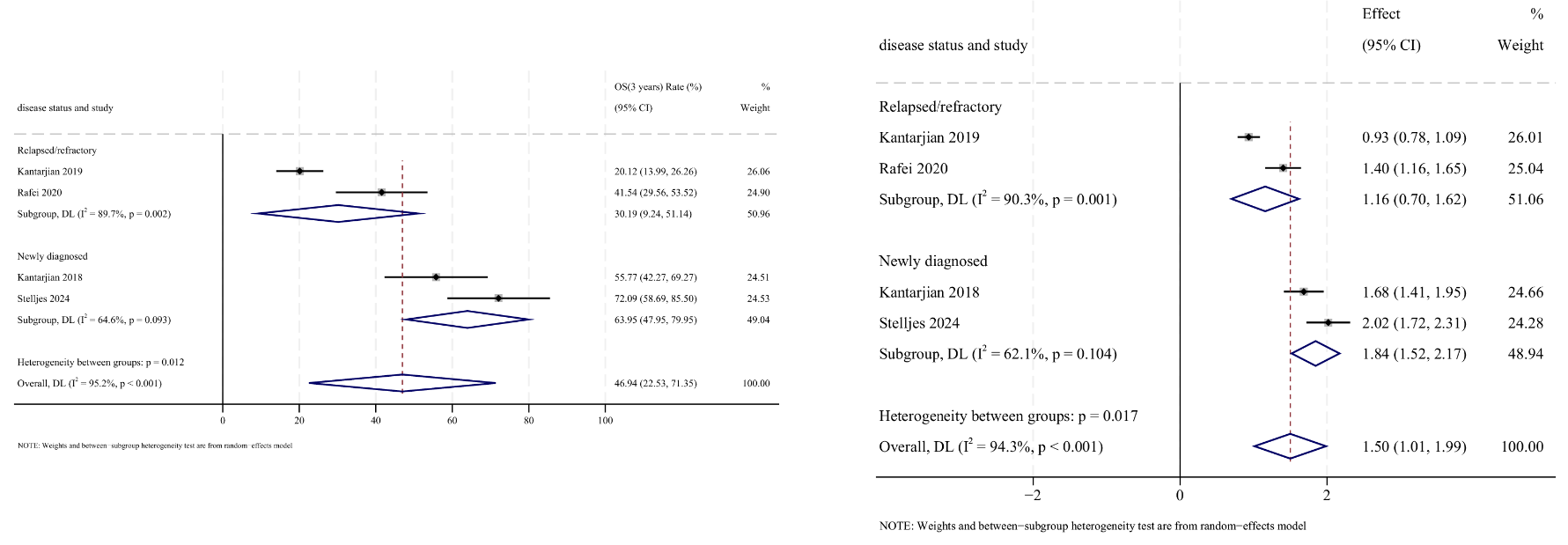


# Figure 4 Forest map of OS（5 years）


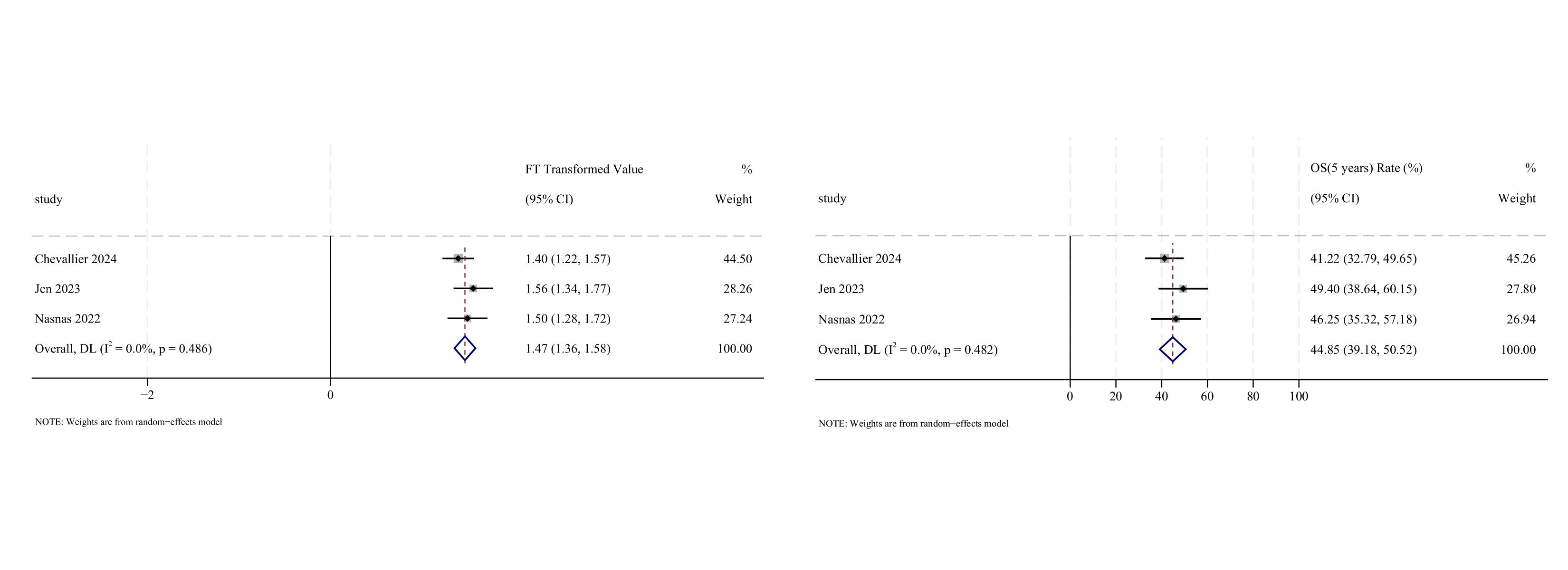

Supplement: Supplementary file 5 [file DataSheet5.docx]
