# Supplementary material for: Efficacy and safety of inotuzumab ozogamicin and its combination therapies in acute lymphoblastic leukemia: a systematic review and meta-analysis
Source: Front Oncol. 2025 Nov 4;15:1613777. doi: 10.3389/fonc.2025.1613777 (PMC12623164; doi:10.3389/fonc.2025.1613777)
Supplement: Supplementary file 6 [file DataSheet6.docx]

| Table 1 Characteristic of included studies. | end points | OR、MRD、HSCT、AEs | OR、CR、MRD、OS、HSCT、AEs | OR、MRD、OS、HSCT、AEs | OR、MRD、OS、AEs | MRD、OS、HSCT、AEs | OR、CR、MRD、OS、AEs |
| --- | --- | --- | --- | --- | --- | --- | --- |
|  | gender male/female | 46/56 | 51/21 | 91/73 | 15/9 | 18/21 | 61/70 |
|  | age | ＞18years | 45years(20-79) | 46.5years(18-78) | 46years(28-76) | 55years（22-84） | 68years(55-84) |
|  | sample size | 102 | 72 | 164 | 24 | 39 | 131 |
|  | intervention | Inotuzumab ozogamicin | Inotuzumab ozogamicin | Inotuzumab ozogamicin | Inotuzumab ozogamicin + EPOCH | Inotuzumab ozogamicin + low-dose chemotherapy | Inotuzumab ozogamicin + low-intensity chemotherapy |
|  | disease status | Relapsed/refractory ALL | Relapsed/refractory CD22+ ALL | Relapsed/refractory ALL | Relapsed/refractory B-cell ALL | Relapsed/refractory Ph- B-cell ALL and Ph+ B-cell ALL | Newly diagnosed CD22+ Ph- B-cell ALL |
|  | follow-up time,months | - | 23.7months（6.8-29.8） | 29.6months（1.7-49.7） | 17.3months（4.9-30.8） | 2.8months(0.6-20.0） | 31.3months（IQR，20.22-37.72) |
|  | experimental period | - | 2011.05.11-2015.1.30 | 2012.8.27-2017.1.4 | 2019.9-2022.11 | - | 2017.12-2022.3 |
|  | study type | open label，non randomized, parallel assignment | open-label, non-randomized phase 1/2 study | RCT | open Label, single-center, non randomized, sequential assignment，phase 1 | multi-center, phase 2A exploratory study | open-label， prospective， multicenter， phase 2 trial |
|  | country | 8 countries | USA | 19 countries | USA | Italy | France |
|  | study | NCT03677596 | DeAngelo et al.(2017) | Kantarjian et al.(2019) | Kopmar et al.(2024) | Marconi et al. (2022) | Chevallier et al. (2024) |
| Table 1 (continued) | end points | OR、MRD、OS、ASCT、AEs | OR、CR、MRD、OS、AEs | OR、CR、MRD、OS、ASCT、AEs | OR、CR、MRD、OS、ASCT、AEs | OR、CR、OS、AEs | OR、MRD、OS、ASCT、AEs |
|  | gender male/female | - | 32/20 | 20/28 | - | - | - |
|  | age | 44years(19-70) | 68years（64-72） | 39years（18-87） | 68years（60-87） | 71years（60-84） | 64years（56-80） |
|  | sample size | 27 | 52 | 48 | 80 | 33 | 43 |
|  | intervention | Inotuzumab ozogamicin | Inotuzumab ozogamicin +low-intensity chemotherapy | Inotuzumab ozogamicin + mini-HCVD, with or without Blinatumomab | Inotuzumab ozogamicin + mini-HCVD, with or without Blinatumomab | Inotuzumab ozogamicin + Blinatumomab | Inotuzumab ozogamicin + chemotherapy |
|  | disease status | Relapsed/refractory Ph- B-cell ALL and Ph+ B-cell ALL | Newly diagnosed Ph- ALL | Relapsed/refractory Ph- ALL | Newly diagnosed Ph-  B-cell ALL | Newly diagnosed CD22+ Ph- B-cell ALL | Newly diagnosed Ph- B-Precursor ALL |
|  | follow-up time,months | 21months | 29months（IQR，13-48） | 31months（1-60） | 61months（7-123） | 22months | 2.7years |
|  | experimental period | 2018.11-2022.6 | 2011.11.12-2017.4.22 | 2012.11-2018.1 | - | - | 2018.6-2021.4 |
|  | study type | open label，single group assignment | open-label, parallel assignment， phase 2 study | open-label, non-randomized, parallel assignment | open-label,non-randomized,phase 2 study | non-randomized， parallel assignment， open label | open-label, single group assignment，phase 2 |
|  | country | USA | USA | USA | USA | USA | Germany |
|  | study | Nasr et al.(2023) | Kantarjian et al.(2018) | Jabbour et al. (2018) | Nasnas et al. (2022) | Wieduwilt et al.(2023) | Stelljes et al. (2023) |
| Table 1 (continued) | end points | OR、CR、MRD、OS、ASCT、AEs | OR、CR、MRD、OS、ASCT、AEs | OR、CR、MRD、OS、ASCT、AEs | OR、CR、MRD、OS、ASCT、AEs | NOTE:OR:over response,CR:complete response,MRD:minimal residual disease,OS:over survival,SCT:Stem cell transplantation,AEs：adverse events，ALL：acute lymphoblastic leukemia，CD22+：CD22-positive，Ph+：Philadelphia chromosome-positive，Ph-：Philadelphia chromosome-negative，EPOCH：edetoposide, prednisone, vincristine, cyclophosphamide, and doxorubi. | |
|  | gender male/female | 27/8.0 | - | 30/35 | - |  |  |
|  | age | 34years（20- 79） | 68years(60-87) | 39years（18-87） | 68years（60-81） |  |  |
|  | sample size | 35 | 83 | 65 | 70 |  |  |
|  | intervention | Inotuzumab ozogamicin | Inotuzumab ozogamicin + mini-HCVD, with or without Blinatumomab | Inotuzumab ozogamicin + mini-HCVD, with or without Blinatumomab | Inotuzumab ozogamicin + mini-HCVD, with or without Blinatumomab |  |  |
|  | disease status | Relapsed/refractory CD22+ ALL | Newly diagnosed Ph- B-cell ALL | Relapsed/refractory ALL | Newly diagnosed Ph- B-Precursor ALL |  |  |
|  | follow-up time,months | 4.4months（0.7-11） | 88months（IQR，41-120） | 36months（1-87） | 45months（2-98） |  |  |
|  | experimental period | - | - | 2013.2-2019.9 | - |  |  |
|  | study type | open-label,non-randomized,phase 2 study | open-label,non-randomized,phase 2 study | open-label,non-randomized | open-label,non-randomized |  |  |
|  | country | USA | USA | USA | USA |  |  |
|  | study | Advani et al. (2014) | Jen et al. (2023) | Rafei et al. (2020) | Short et al. (2020) |  |  |
